# Strategic Encounters in Innovation and Regulation: Healthcare Transformation in the Era of Digital Connectivity: Comment on "What Managers Find Important for Implementation of Innovations in the Healthcare Sector – Practice Through Six Management Perspectives"

**DOI:** 10.34172/ijhpm.2022.7271

**Published:** 2022-06-07

**Authors:** Sandro Mendonça, Bruno Damásio, Fernando Santiago, Martin Chen, António Bob Santos, Miguel Pina e Cunha, Antonio Nicita

**Affiliations:** ^1^BRU-IUL and ISCTE Business School, Lisbon, Portugal.; ^2^REM-UECE/ISEG, University of Lisbon, Lisbon, Portugal.; ^3^SPRU, University of Sussex, Brighton, UK.; ^4^ANACOM, National Communications Authority for Portugal, Lisbon, Portugal.; ^5^NOVA Information Management School (NOVA IMS), Universidade Nova de Lisboa, Campus de Campolide, 1070-312 Lisboa, Portugal.; ^6^UNIDO, Vienna, Austria.; ^7^Links Medical Scientific, Co. Ltd., Irvine, CA, USA.; ^8^DireçãoGeral do Ensino Superior, Lisboa, Portugal.; ^9^Nova School of Business and Economics, Lisbon, Portugal.; ^10^Università di Roma LUMSA, Roma, Italy.

**Keywords:** Strategic Encounters, Innovation Management, Regulation, Healthcare

## Abstract

Healthcare innovations emerge and develop in institutionally dense selective environments. New projects and propositions in healthcare sectoral ecosystems can be understood as product-service compacts, that is, complex solutions that dynamically integrate tangible and intangible elements in close interaction with users’ needs and the evolving regulatory context under uncertainty and ambiguity. We advance the concept of "strategic encounters" to encapsulate, capitalise and extend the contribution by Palm and Fischier’s on the key enabling managerial factors for healthcare innovation implementation under conditions of imperfect foresight. We intertwine creative assemblages that shape the formation of knowledge-intensive activities at the operators’ level with scope of sectoral level interventions to underscore how the opportunities and constraints can enhance innovation for the common good. We use the case of digital data health regulatory agendas as illustration. We argue that this broader perspective on healthcare transformation is theoretically pertinent and practically useful, for management and policy.

## Introduction

 When studying innovation – or organizing, in general, one might say – theory is nothing; theorising is everything. To paraphrase as Mike Tyson: “you have an innovation strategy until you get punched in the face.” In actual change as it takes place on the ground (or in the digital sphere), and in contrast with broad-brush abstraction, micro-processes do matter. Asserting this assumption is to make the case for innovation implementation as an occasion in which generative deviation according to circumstance (vicarious innovation) can be capitalised upon (organisational learning) for the sake of furthering knowledge-seeking activities (high-tech industries or sophisticated services) in presence of over-abundant ambiguity and uncertainty (when information is imperfect and feedback from the environment difficult to interpret).

 The context through which new ideas, solutions, protocols and devices must navigate is dense and dynamic. When new projects and novel business case proposals are introduced, they face persistent pushes and pulls as well as situational stresses and synergies. How innovation mutates and adapts is contingent on the complexities of the evolving environment. To appreciate innovation as process in real-time is to be aware of the significance of its open-ended nature but also of the dialectical frame in which it is nurtured. Innovation is therefore a surprise-based activity that negotiates capabilities and navigates constraints.

 By emphasising implementation as a *transformative movement,* we ask why and how “doing and becoming” fuse so to release the potential (and explain the persistence) of innovation. This is equivalent to referring to innovation in a “problem-solving” light. However, “problem-solving” is a mode of learning predicated on agile organizations with routines that are both flexible and oriented towards timely outcomes, with the outcomes themselves have metrics that are clear, transparent and can be monitored and enforced. In this contribution, the proposal is to turn such problem-solving view around, that is, innovation is here seen not as a tension-resolving task but as a generative practice from which new paradoxical tensions emerge. This view, which stresses the value of real-time learning, draws attention to the particulars of implementation as an angle of analysis. By doing so we bring to the fore a set of salient factors that Palm and Fischier^1^ have recently identified, on which this paper comments and adds complementary considerations (including from the policy angle).

 In this regard, the notion of “strategic encounters” could promise some concise conceptual elicitation of *innovation as a paradox*, ie, as mutually defining persistent oppositions.^2^ The concept of the “strategic encounters” is defined here as the productive direct contact of an innovation with the selective ecosystem, including the serendipity resulting from immediate face-to-face interactions with users and the mediating feedback mechanics with institutions. Innovation is thus seen as outcome of strategic encounters triggered through the practicality of actual challenges (ie, the *transformative movement*), rather than a mere totemic presence (ie, the cult of an innovation drive in the organizational community).

 In this article, we focus on healthcare innovation as an exemplary application of the notion of “strategic encounter.” Healthcare innovations emerge and develop, are introduced and diffused, encouraged and strained by very specific sectoral systems of innovation.^3^ They can be best understood as product-service compacts, that is, integrated packages of tangible and intangible components that are shaped by both the *hard* and *soft* knowledge-bases of innovators in a context of imperfect foresight. What happens when healthcare innovations face (and react to) resistance in the institutional and regulatory context is what matters for our limited purposes here. We bring to the fore healthcare digital innovation as an example for analysis.

## Innovation Is Situated Sectoral Conversation

 Health enhancement and disease elimination is a multidisciplinary undertaking. It depends on science and technology, but also on engaging many publics and stakeholders, namely the professionals and the patients, as well as the national agencies and sectoral regulators responsible, among other things, for ensuring observance of agreed ethical ways of conduct governing the use or not of certain approaches to healthcare provision.^4^ In this realm, there is competition and cooperation, disputes and disagreements, consensus and convergence. In other words, there are tensions with paradoxical features, persisting but potentially productive if adequately harnessed. Knowledge and persuasion are involved: discovery and technique do not speak for themselves, they need interpreters and advocates.^5^ For instance, more advanced medical treatments need to be connected to standardising bodies, accepted by the expert communities, desired and picked by patients (and their families), etc. Hence, innovation is a *positive-normative combination*, ie, it is equivalent to stating a hypothesis of how things are and how things ought to be.

 Innovation tensions take place at different levels. Moreover, innovation has a consequential non-market dimension. The process is participative and plural by design, having a great deal of direct and indirect involvements. Neo-Schumpeterian scholars share the view that creation in a dynamic sector depends on: (*i*) *knowledge base and technological trajectories*, as sources of opportunities; (*ii*) *agents and networks*, as drivers of collaboration and change; (*iii*) *institutional infrastructure*, as structural filters and selectors. In other words, the sectoral system locates innovation processes in inner learning routines, actor behaviours and interactions, and the institutional and regulatory context. These observations are consistent with literature that places innovation, including in healthcare, as being determined by and shaping institutional variation, that is, the institutions drift shaping learning and capability building pathways.

 When researchers and managers conjecture and prepare to launch new offerings, sometimes the phase of implementation receives less attention (an important insight by Palm and Fischier^1^). In the field of healthcare, however, the actual details and instituted filters are significant. These filters are resource-consuming (regulatory structures are expensive to maintain and impose administrative costs) and the regulatory constraints are not merely technical in nature (they represent the interests of the broader societies and the asymmetries of non-unbiased political economies). As original innovation proposals get into contact with market realities and regulatory controls, a new set of stimulus takes place and the dynamics that unfolds affects the original intent and reach of its promoters.

## Strategic Encounters Define Innovation as a Transformative Movement

 Discovery and knowledge accumulation pathways are hit by external events, which may be noisily pre-announced by weak signals or entirely be unforeseen wild cards.^6^ Ambiguity permeates innovation processes and disruption punctuates as learning develops through dense and dynamic environments. This sometimes forces organisations to draw to much attention to short-term tactics. As General Dwight Eisenhower put it: under the endless flow of incidents and the occasional unprecedented crises “operations will eat-up the long-range planning” (p. 6).^7^

 In conditions of shifting and kaleidoscopic uncertainty, accidental findings and connections (as well as entropy and decay) happen all the time. So, paradoxically, the fog of ignorance is constitutive of innovation management and serves as an input to technical creativity. Not only errors are fundamental ingredients in discovery, but also unexpected encounters are (with users, regulators and other stakeholders). That the unexpected can be harnessed for purposeful consistent action also means that rationality can be rescued from itself, making viable evolutionary strategizing as an explicit intellectual project. The recognition of the possibilities and contradictions of bricolage is necessary to rebuild an apt architecture for strategic decision-making.^8^ Thus, intended and unintended encounters become a force for production and renewal. Essentially this means that innovation incorporates emergence and eventfulness; “best way” modes of organising are slippery and dangerously illusory in the context of processes rich in serendipity.^9^ Considering “strategic encounters,” ie, the critical junctures that are bound to happen when advancing through chain-linked problems, helps to bring out the strategic from the tactical instantiations in decision-making.^10^

 Continuous motion and effectual assemblages take place in precarious spaces with various layers of complexity (on the verge of chaos) where tentative solutions (on the verge of chaos) become better problems (happy surprises, serendipitous discoveries) that provoke further self-subversion (what we call here *transformative movement*). The role for organising and leadership consists in making sense and directing luck and chance, either in private managerial work (in firms and industry associations) or public duties (government policy and independent regulation). Thus, acknowledging and promoting “strategic encounters” allows for innovating something out of something else. The final outcome will depend both on the modality of matching and on the identity of matchers. This posture fuses *anticipation* and *improvisation*, synthesizing *doing* with *becoming* and *strategizing* with *executing*.

## The Importance of Actual Innovation Implementation in The Healthcare Sector

 Palm and Fischier^1^ bring this perspective into sharp focus by showing how dealing with obstacles is crucial as a specific step in moving from idea generation to innovation implementation in the healthcare sector. From these authors, we come to see management and leadership as the process of creating internal leeway for innovation and the need to deal with the external ecological frame that shapes its actual practice.

 Following Palm and Fischier,^1^ the single most important challenge for management is to create a holistic image in which space for innovation is enabled and enlarged. The analytical framework they proposed is a six-factor list that we can decompose in three sub-sets:

- resources and routines (*Resource availability* and *Human capital management*);
- organisational architecture (*Organisational culture* and *Organisational structure*); and
- interactive competence (*Collaboration with the beneficiaries for the healthcare effort* and *Collaborations with other relevant stakeholders*).

Their contribution points out that the presence of some elements (libraries of knowledge assets, encouragement of small-scale prototyping, etc) is fundamental for innovation, but also that fit and feedback (continuous investment in relating to the values of users, open and circular iterative processes) are crucial for constructive visions for the future. In a word, Palm and Fischier underscore the importance of the active creation of circumstances under which it becomes possible for healthcare innovators to implement solutions. In a complementary fashion, our own point is that in dense and dynamic environments these solutions themselves lead to “strategic encounters,” ie, the endogenously-derived but unpredictable opportunities (hence “encounters”) to discover and address new and better problems that are meaningful beyond their short-term instantiations (hence “strategic”).

## From Management to Policy: And the Case of Digital Health Regulation

 Grand challenges that impact the health sector constitute a wake-up call for public intervention, ranging from government policy to regulatory action. Major urgencies and emergencies such as the digital transition, climate change, pandemic crises, and geopolitical raptures constitute a focusing device that impels to retain these lessons and enact active strategies for implementing comprehensive and coordinated policies. How can macro-level strategists influence the manoeuvring space of managers on the ground?

 It is known that healthcare providers, medical equipment manufacturers, and specialised knowledge-based consultants need to deliver product-service combos, to integrate information and communication technologies into their business models, and continuously optimise investment while safeguarding credibility and reputational capital, especially in cross-border/cross-regulatory settings. The role of frameworks-setters (policy-makers, supervising authorities, etc) can be two-fold. First, the scope and the scale, the agility and the endurance of the framework shapers are thus instrumental in nudging the evolution of sectoral systems of innovation as a whole (see Box 1), by enabling and framing the possible directions in which serendipity can take place. Second, moving the local experiments/experience of the individual operator to the national/international level is a major way for achieving inclusive changes and the common good.

**Box 1. Digital Innovation and Regulatory Framing in Healthcare**

The announcement of the “Healthy China 2030” blueprint marks another milestone in the history of the country’s healthcare reform. It is a signal that China would put health at the centre of the country’s entire policy-making strategy. In a long-term perspective, it extensively covers areas such as medical services, insurance, food and drug safety, active ageing, physical exercise, thus indicating that reform is not limited to the diagnosis-treatment nexus but integrates the broad determinants of health (more than the absence of disease) and wellbeing (phycology, physiology, environment).

By embedding different players (from the medical, pharmaceutical, financial fields), the central government authorities aim to resolve the mismatches in the health system.^11^ Smart health capabilities from the supply side (eg, wearable, data, robotics, 5G, etc) and empowered users from the demand side (eg, self-checks through home testing devices, quality online experience, digital family doctors as expert consultants and coaches, emphasis on emotional health) are two pillars raising the standard. Under the aegis of the “Internet Plus” strategy, digital technologies and management styles are indeed being used to wire up capabilities and needs. Examples of the effective overcoming of some traditional barriers through integrated digital cooperative approaches are “community clinics” and “internet hospitals,” but there plenty of competitive approaches, like mobile medical platforms (Doctor 7LK, Doctor Xingren, Micro-doctor, Doctor Hao, Doctor Chunyu, etc).^12^

The lively experimentation mode of healthcare innovation in China is framed by many adaptive regulatory schemes, some of which are innovative and themselves on trial implementation: “Administrative Regulations on Telemedicine Services,” “Administrative Measures on Standards, Security, and Services of National Healthcare Big Data,” “Medical Devices Regulations,” etc. Key Chinese regulatory authorities are: National Health Commission, National Medical Products Administration, National Healthcare Security Administration, State Administration for Market Regulation, among others. Chinese regulatory style is experimental, it displays fluid and flexible governance under hierarchy, and shows itself to be open to creative accumulation as reform builds on reform.^13-16^

## Conclusion

 New projects, novel value-propositions, business model innovations in healthcare sectoral ecosystems can be best understood as *product-service compacts*, that is, complex solutions that dynamically integrate tangible and intangible elements in close interaction with users’ needs and the evolving regulatory context. This paper advanced the concept of “strategic encounters” to encapsulate, capitalise and extend the contribution by Palm and Fischier^1^ who stress actual implementation of innovation and refer to key enabling managerial factors in the healthcare case. We do this by bringing more explicitly the role of dense and dynamic environments in shaping ongoing innovation. We call attention to the paradoxical integration of opposites such as planning and improvising, while preparing for and accepting serendipity.^2^ By highlighting societal challenges, like digital health regulation, we have considered how opportunities and constraints weave together in a way that is also relevant for framework-setters (ie, meso and macro-level actors like government agencies). We believe this broader perspective on healthcare transformation is theoretically pertinent and practically useful, both for management and policy.

## Acknowledgements

 Sandro Mendonça acknowledges support by FCT (Fundação para a Ciência e a Tecnologia), Portugal, by the Business Research Unit (BRU-IUL) and by UECE-REM (Research Unit on Complexity and Economics). BRU-IUL also benefited from grants UID/GES/00315/2013, UIDB/00315/2020; UIDB/05069/2020; PTDC/EGE-ECO/30690/2017 and is part of the project PTDC/EGE-ECO/30690/2017. Sandro Mendonça concluded the paper while he was Visiting Professor at the Dept. of Econoimics of the University of Insumbria, Italy, and he thanks the institution for the research context and support.

 Bruno Damásio acknowledges the financial support provided by FCT under the project UIDB/04152/2020 - Centro de Investigação em Gestão de Informação (MagIC). Martin Chen acknowledges the research context provided by the doctorate in management developed between ISCTE-IUL, Portugal, and the Southern Medical University, China. Miguel Cunha acknowledges funding by FCT (UID/ECO/00124/2019, UIDB/00124/2020 and Social Sciences DataLab, PINFRA/22209/2016), POR Lisboa and POR Norte (Social Sciences DataLab, PINFRA/22209/2016). Sandro Mendonça and António Nicita have currently regulatory duties, but they are here as authors in a personal capacity alone. All shortcomings are attributable to the authors alone.

## Ethical issues

 Not applicable.

## Competing interests

 Authors declare that they have no competing interests.

## Authors’ contributions

 Conception and Design: SM, BD. Drafting of the manuscript: SM, BD, FS, MC, ABS, MPC, and AN. Critical revision of the manuscript for important intellectual content: SM, BD, FS, MC, ABS, MPC, and AN.
